# The efficacy of subglottic secretion drainage in preventing ventilator-associated pneumonia in the intensive care unit: a systematic review and meta-analysis

**DOI:** 10.3389/fmed.2026.1758199

**Published:** 2026-04-15

**Authors:** Hengfen Hu, Rexidan Tursuniyazi

**Affiliations:** 1 Medical School, Hunan Polytechnic of Environment and Biology, Hengyang, China; 2 Tulufan Vocational Technical College, Tulufan, China

**Keywords:** ICU, meta-analysis, nursing, subglottic secretion, ventilator-associated pneumonia

## Abstract

**Objective:**

To review and analyse the clinical nursing effect of subglottic secretion drainage (SSD) on patients with intensive care unit (ICU) ventilator-associated pneumonia (VAP) through a systematic review and meta-analysis.

**Methods:**

The clinical articles published between January, 2000 and May, 2024 were screened from PubMed, Web of Science, Spring, and Science Direct databases. After that, the quality of selected articles was assessed and relevant data were extracted.

**Results:**

According to different adjuvant mechanical ventilation (MV) methods of ventilator, the included patients were enrolled into SSD and control groups. The patients in the former group were treated with SSD, while those in the latter group received routine treatment. RevMan5.3 was employed for the meta-analysis of relevant data. Next, the incidence of VAP, MV duration, length of stay in ICU and hospital, fatality, tracheostomy, incidence of VAP/1,000 ventilator days, and the detection rates for Gram positive and negative bacteria among patients in the two groups were compared. According to the outcomes, 14 qualified articles were included. In contrast to those in control group, the incidence of VAP (OR = 0.42, 95% CI: 0.29 ~ 0.61; *Z* = 4.52, *p* < 0.00001), VAP/1,000 ventilator days (OR = 0.44, 95% CI: 0.29 ~ 0.66; *Z* = 3.91, *p* < 0.0001), and the detection rates for Gram positive and negative bacteria (OR = 0.41, 95% CI: 0.25 ~ 0.68; *Z* = 3.44, *p* = 0.0006; OR = 0.64, 95% CI: 0.46 ~ 0.90; *Z* = 2.57, *p* = 0.01) all remarkably declined and length of ICU stay time (mean difference (MD) = −0.47, 95% CI: −0.90 ~ −0.04; *Z* = 2.13, *p* = 0.03) was shortened in SSD group.

**Conclusion:**

The findings revealed that SSD could notably reduce the incidence of VAP and the detection rates for Gram positive and negative bacteria and shorten length of ICU stay time.

**Systematic review registration:**

INPLASY202630098 (INPLASY.COM).

## Introduction

Ventilator-associated pneumonia (VAP) is a common acquired infection during mechanical ventilation (MV) treatment in intensive care unit (ICU) (1). It refers to parenchymal lung inflammation occurring within 48 h after the aspiration of bacteria into lower respiratory tract caused by the retention of massive secretions on the surfaces of trachea and saccus 48 h after MV treatment or the termination of MV and extubation among patients without pulmonary infection (2). Drug resistance is prevalent in VAP pathogenic bacteria, which causes difficulties in VAP and increases the incidence and fatality of VAP (3). According to relevant data, the incidence of VAP in ICU ranges from 8 to 28% and the fatality ranges between 25 and 76% (4). The mortality among patients with ICU and VAP rises by 1 to 9 times (5). According to relevant studies, VAP attributable mortality is close to 30%, relative risk (RR) of death amounted to 2.0 (6), ICU duration was prolonged by 4.3d, and death risk increases by 5.8% (7). The occurrence of VAP prolongs the duration of MV and hospital stay and increases medical costs and death risk (8, 9). Hence, effective and timely prevention and treatment of VAP in ICU is of great significance for the improvement of prognosis.

Subglottic secretion drainage (SSD) is an operation technique of negative pressure drainage of the secretions on saccus with the drainage channel attached to tracheal wall. It is a new method for preventing VAP (10). The main SSD methods include continuous aspiration of subglottic secretion (CASS), intermittent aspiration of subglottic secretion (IASS), and subglottic rinsing. CASS and IASS are mainly adopted in clinical practice. During CASS, the secretions remaining on saccus were drained and flushed to prevent pulmonary infection caused by aspiration and reduce the incidence of VAP and the retention time of subglottic residue on the saccus (11, 12). Walaszek et al. (13) showed that RR increased by 7.5 times without the treatment for MV patients with SSD. Multiple research findings demonstrated that SSD could effectively reduce VAP incidence and shorten MV duration in contrast to traditional endotracheal aspiration (14, 15). Nonetheless, some research demonstrated that SSD caused mucosal injury, falling off of cilia in tracheal mucosal epithelial cells, hemorrhage, insufficient suction, and drainage tube blockage and increased the risk of infection. As a result, the incidence of VAP rose (16).

Based on the above research findings, the clinical therapeutic effect of SSD on VAP nursing for patients undergoing ICU treatment is apparently controversial. Therefore, a meta-analysis was performed to systematically assess the clinical efficacy of SSD on VAP nursing in ICU to provide some references and basis for the prevention and treatment of VAP.

## Materials and methods

### Data inclusion methods

A thorough search was independently conducted by two researchers across multiple databases, including PubMed, Embase, Cochrane Library, and Web of Science. Critically ill patients who needed to undergo MV through oral or nasal tracheal intubation or tracheal incision were selected as the subjects. The screened articles were randomized controlled trials (RCTs) and the included interventions included all SSD methods. The included data mainly included titles, the first authors, publication year, number of cases, grouping, the number of cases in each group, interventional methods for different groups, and observation indicators. Inclusion and exclusion criteria.

The inclusion criteria were as follows:

- Critically ill patients aged 18 and above.
- Patients treated with MV for 48 h or longer.
- The interventional method for experimental group was one of SSD methods, including CASS, IASS, and subglottic rinsing. The patients in control group received routine MV nursing.
- The included articles were published between January, 2000 and May, 2024.
- The included articles were RCTs.
- Outcome indicators were recorded in detail and completely, including VAP incidence, the duration of MV and stay in ICU and hospital, the mortality in ICU, mortality, tracheostomy, mean cuff pressure, incidence of VAP/1,000 ventilator days, the detection rates for Gram positive and negative bacteria, positive end-expiratory pressure (PEEP), and the incidence of reintubation.

The exclusion criteria were as follows:

- The articles with incomplete data.
- Literature reviews, expert commentary, editor comments, news reports, and product description.
- The articles with unknown MV time before entering ICU.
- The articles without outcome indicators.
- The articles were non-RCTs.
- The articles were published repeatedly.
- Patients were gravidas and suffered from mental disorders.
- The articles without SSD treatment for various reasons.
- Animal experiments, *in vitro* cell experiments, and other fundamental research.

### Retrieval strategies

The articles published between January, 2000 and May, 2024 were retrieved from PubMed, Nature, Web of Science, Spring, and Science Direct and other online databases. The key search terms included “subglottic suction,” subglottic secretion drainage,” “aspiration of subglottic secretion,” “pneumonia,” “ICU,” “ventilator,” “SSD,” “ventilator associated pneumonia,” “VAP,” “continuous aspiration of subglottic secretion,” “CASS,” “intermittent aspiration of subglottic secretion,” “IASS,” “Intermittent subglottic drainage,” “ISD,” and “mechanical ventilation.” They were connected using “or” and “and” for combined search. MeSH subject term was combined with free term for the retrieval.

### Article screening and quality evaluation

The retrieval results were imported into EndNote article management software. The articles were independently screened by 2 reviewers according to the inclusion and exclusion criteria. The disqualified and low-quality articles were excluded and the remaining articles were preliminarily screened by reading titles. The missing data should be supplemented by contacting the first authors. After that, abstracts and full texts were further read to select the qualified articles. The disagreement on the review result should be resolved through the discussion between 2 reviewers or the final assessment with a third reviewer. All usable variables were extracted from the selected articles and then input into Microsoft Excel database.

Cochrane Reviewer’s Hand-book 5.1.0 bias risk evaluation standard was utilized to evaluate the quality of screened articles. The specific contents of the evaluation standard were as follows:

- Whether research methods were correct and comprehensible.
- Whether the generation of random sequence was clarified in the articles.
- Whether research findings were clear and unambiguous.
- Whether there was selective reporting in the articles.
- Whether there were blind controlled studies on participants and personnel in the articles.
- Whether research results were evaluated using blind method.
- Whether data were complete and there was selective reporting in the articles.

According to the Cochrane Risk of Bias tool, studies were evaluated across seven domains and categorized as having a ‘low’, ‘high’, or ‘unclear’ risk of bias. Two reviewers independently assessed the studies and performed a cross-check. Disagreements were resolved by a third reviewer.

### Data extraction

The data of screened articles were extracted by 2 reviewers, mainly including basic data (titles, the first authors, publication year, and publication periodicals), subjects (included research objects, grouping, the number of cases in different groups, and breeding methods, age, and gender of patients in different groups), and outcome indicators (VAP incidence, the duration of MV and stay in ICU and hospital, fatality, tracheostomy, incidence of VAP/1,000 ventilator days, and the detection rates for Gram positive and negative bacteria).

### Statistical methods

Excel 2016 was utilized to sort out the data in screened articles and Cochrane Reviewer’ Handbook was used to evaluate the quality of articles. What’s more, RevMan5.3 was employed for the meta-analysis. Enumeration data were denoted by RR and 95% confidence interval (CI) and measurement data were expressed as mean difference (MD), standard deviation (SD), and CI.

Heterogeneity analysis was carried out as follows: Firstly, chi-square test was performed to preliminarily identify the heterogeneity of articles. The significance level was set as *α* = 0.05 and *p* < 0.05. After that, the testing result was quantitatively assessed using *I*^2^ in RevMan5.3. *I*^2^ < 25, 25% < *I*^2^ < 50%, and *I*^2^ > 50% suggested low heterogeneity, moderate heterogeneity, and substantial heterogeneity, respectively. Based on the results, fixed effect model was employed for the meta-analysis if *I*^2^ < 50%, while random effect model was utilized if *I*^2^ > 50%. RevMan5.3 was employed to draw funnel plots for the analysis of potential publication bias and forest plots were output. Next, values *Z* and *P* were extracted from the results for the judgment of the meta-analysis results. Sensitivity analysis was carried out by excluding the articles with the poorest quality. Inverted funnel plots were displayed through funnel plots for the observation on potential publication bias. *p* < 0.05 suggested that the differences between groups revealed statistical significance.

## Results

### Article retrieval process

**Figure 1:**
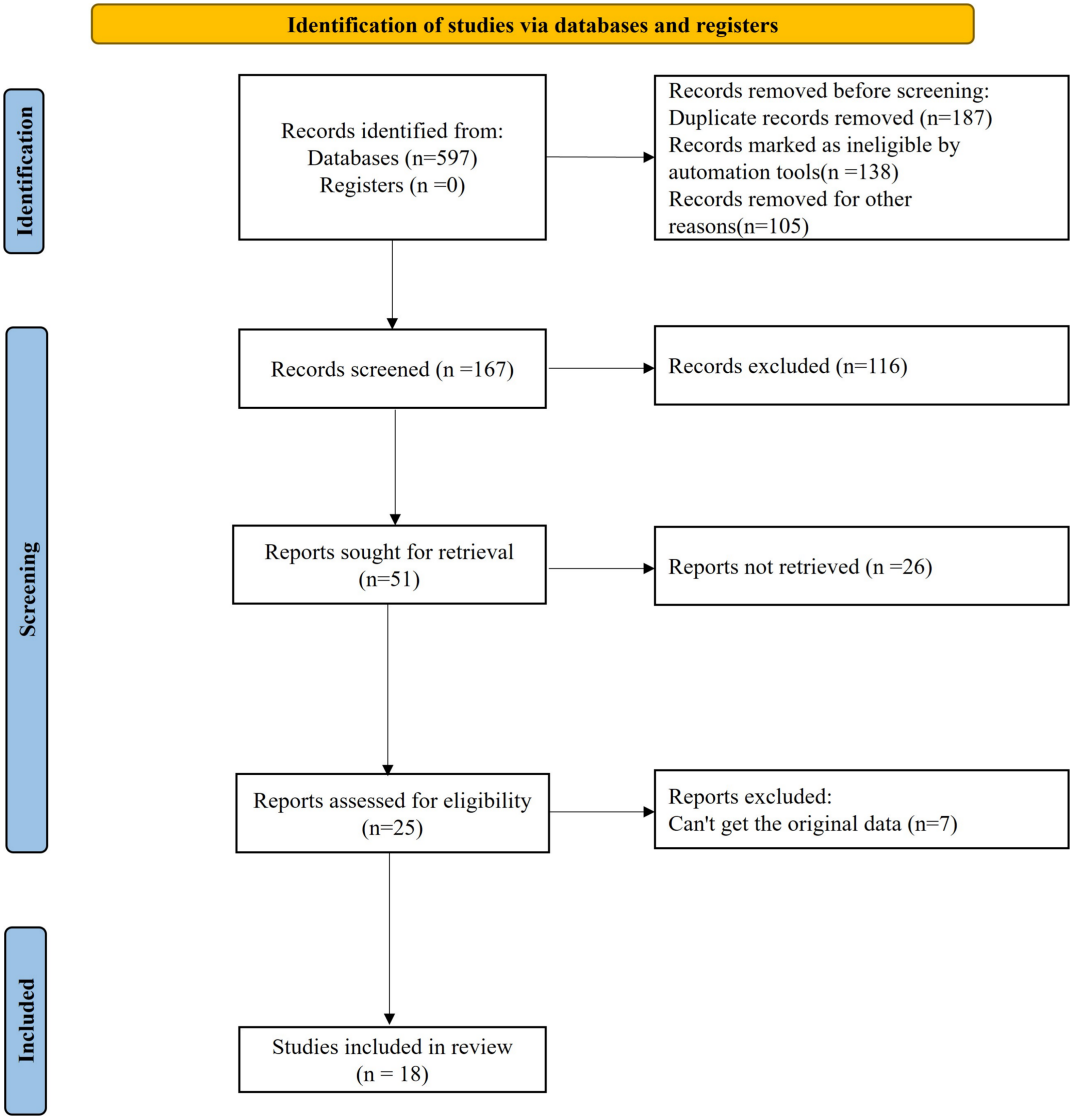
Processes of article retrieval and screening.

A total of 597 articles were initially identified through the database search according to the aforementioned retrieval strategies. After preliminary screening, 187 duplicate articles were deleted, 138 articles marked as ineligible by automated tool were recorded, and 105 articles were eliminated for other reasons. Finally, 167 relevant articles were included. After ineligible articles were removed based on titles, 51 articles were collected. After that, reviews, meeting summary, case analysis, and risk factor assessment were excluded by reading abstracts and research contents. After the preliminary screening, 25 qualified articles were collected. After the further intensive reading, 7 articles with inaccessible original data were removed and 18 were eventually included. The processes of article retrieval and screening were displayed in Figure 1.

### Basic data of screened articles

Eventually, 18 articles and 10,990 subjects were included. Among all included subjects, 4,696 patients received SSD intervention, while the remaining 6,294 patients underwent routine MV nursing. The basic data of screened articles were presented in Table 1.

**Table 1:** Basic data of screened articles.

The first authors	SSD group	Control group	Interventions for SSD group	Interventions for control group	Outcome indicators
Bouza (31)	331	359	CASS	Not undergo CASS	①②③④⑤⑦⑧⑨
Chen (32)	39	42	SSD	No intervention	①②③⑤⑥
Damas (33)	170	182	SSD	Not undergo SSD	①③④⑤⑥
Erinc (34)	12	30	SSD	Conventional	①②③⑤⑦⑧⑨
Girou (35)	8	10	CASS	standard care	①②⑧⑨
Hudson (36)	2,450	2,430	CASS	Routine postoperative medical care	①②③④⑤⑥
Juneja (37)	60	78	ISD	No intervention	①②③④⑦
Lacherade (38)	169	164	SSD	Not undergo SSD	①②③⑤⑥⑦⑧⑨
Lorente (39)	140	140	SSD	Conventional endotracheal	①②③⑥⑧⑨
Millot (40)	55	45	SSD	Standard tracheal	①②③⑥
Nam (41)	468	2,108	SSD	No intervention	①②③⑤⑥⑦
Safdari (42)	38	38	SSD	Routine oropharyngeal suctioning	①
Smulders (43)	75	75	SSD	Standard endotracheal	①②③④⑤⑦⑧⑨
Terragni (44)	232	125	SSD	Not undergo SSD	①③
Kollef (45)	160	183	CASS	routine oropharyngeal suctioning	①②③④⑤
Vallés (46)	76	77	CASS	ROUTINE oropharyngeal suctioning	①②③⑤⑧⑨
Mahul (47)	75	70	SSD	Not undergo SSD	①⑤
Mahmoodpoor (48)	138	138	ISD	No ISD	①②③④⑤

① Incidence of VAP, ② MV duration, ③ duration of stay in ICU, ④ duration of hospital stay, ⑤ fatality, ⑥ tracheostomy, ⑦ incidence of VAP/1,000 ventilator days, ⑧ Gram positive bacteria, ⑨ detection rate for negative bacteria.

**Figure 2:**
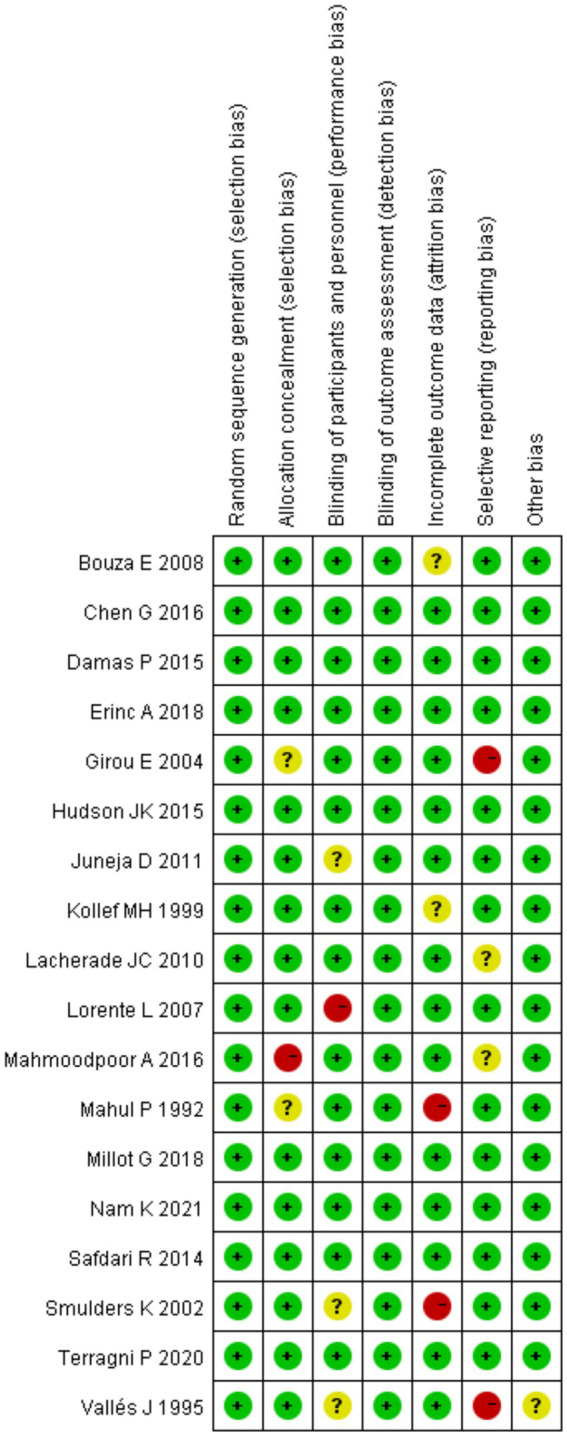
Summary of the assessment of bias risk of included articles.

**Figure 3:**
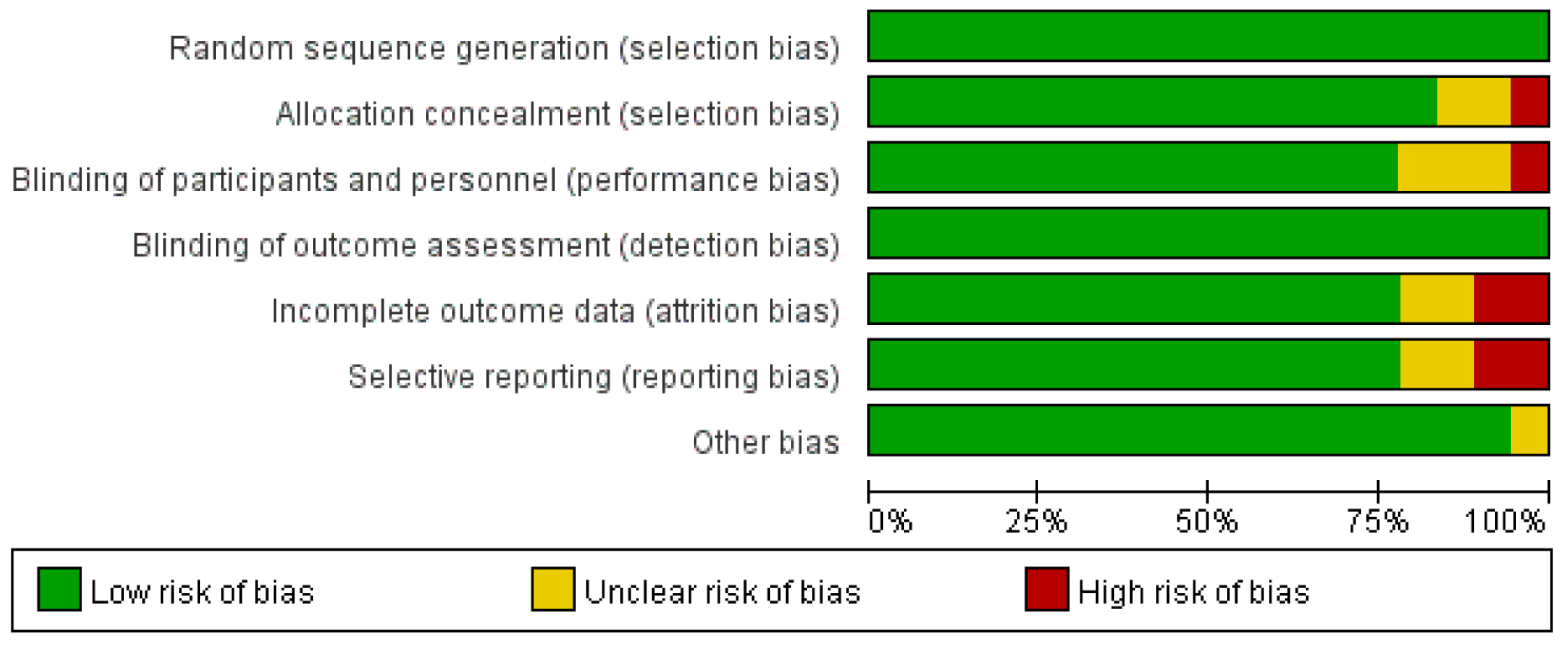
Bar charts of the assessment of bias risk of screened articles.

Evaluation of the quality of included articles Cochrane Reviewer’ Handbook was employed to assess the quality of 18 screened articles. Besides, RevMan5.3 was utilized to draw the summary and bar charts of the assessment of bias risk (Figures 2, 3).

### Comparison of the incidence of VAP between SSD and control groups

**Figure 4:**
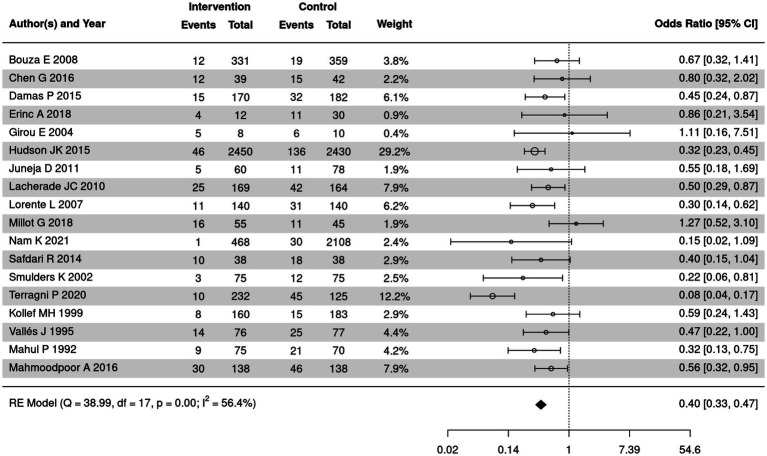
Forest plot of VAP incidence between SSD and control groups. CI, confidence interval; OR, odds ratio.

VAP incidence of SSD and control groups was summarized in 18 included articles. The meta-analysis was implemented to compare VAP incidence among patients in different groups (Figure 4). Significant heterogeneity was detected among the included studies (*I*^2^ = 56.4%, *p* < 0.05); therefore, a random-effects model was employed. The meta-analysis demonstrated that the incidence of VAP in the SSD group was significantly lower than in the control group (OR = 0.40, 95% CI: 0.33–0.47, *p* < 0.00001).

### Comparison of MV duration between SSD and control groups

**Figure 5:**
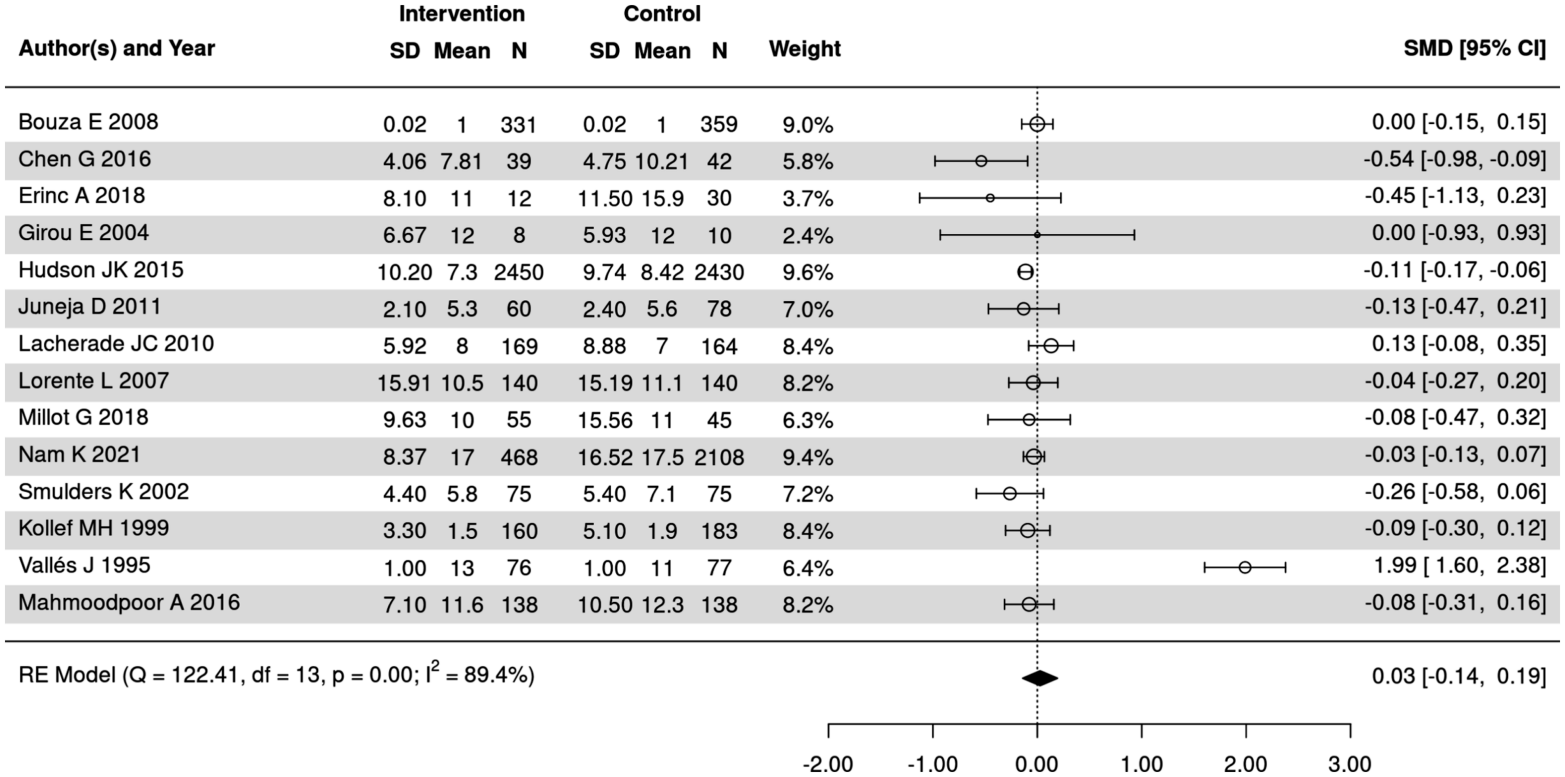
Forest plot of MV duration between SSD and control groups. CI, confidence interval.

MV duration in SSD and control groups was summarized in 14 included articles. The meta-analysis was performed to analyze the difference in MV duration in the two groups after intervention (Figure 5). Significant heterogeneity was detected among the studies (*I*^2^ = 89.4%, *p* < 0.05); therefore, a random-effects model was used. The results showed no significant difference in MV duration between the SSD and control groups (SMD = 0.03, 95% CI: −0.14 to 0.19, *p* = 0.74).

### Comparison of length of ICU stay between SSD and control groups

**Figure 6:**
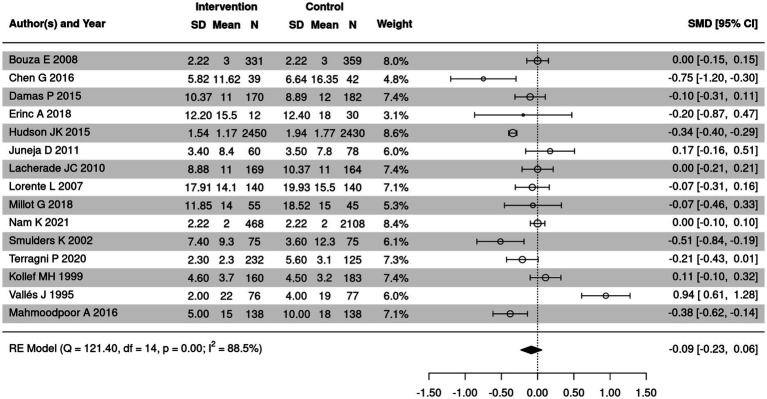
Forest plot of the comparison of length of ICU stay in SSD and control groups. CI, confidence interval.

Length of ICU stay of patients in SSD and control groups was summarized in 15 out of 18 included articles. The results of heterogeneity test were displayed in Figure 6. Significant heterogeneity was detected (*I*^2^ = 88.5%, *p* < 0.05); therefore, a random-effects model was employed. The meta-analysis revealed no statistically significant difference in the length of ICU stay between the two groups (SMD = −0.09, 95% CI: −0.23 to 0.06, *p* = 0.25).

### Comparison of length of hospital stay in SSD and control groups

**Figure 7:**
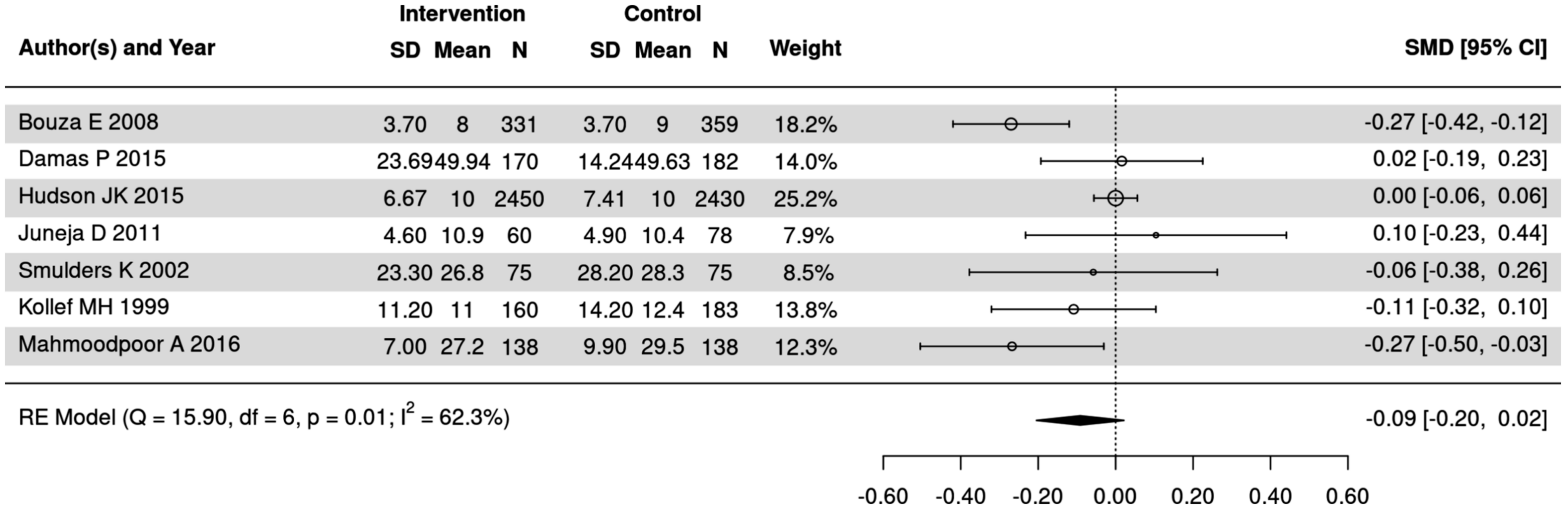
Forest plot of length of hospital stay in SSD and control groups. CI, confidence interval.

Length of hospital stay of patients in SSD and control groups was summarized in 7 out of 18 included articles. According to the results of heterogeneity test, heterogeneity was detected in length of hospital stay in the two groups (*I*^2^ = 62.3%) (Figure 7). Therefore, random effect model was utilized for meta-analysis. It was demonstrated that no apparent heterogeneity was detected in the two groups (SMD = −0.09, 95% CI: −0.20 ~ 0.02).

### Comparison of fatality in SSD and control groups

**Figure 8:**
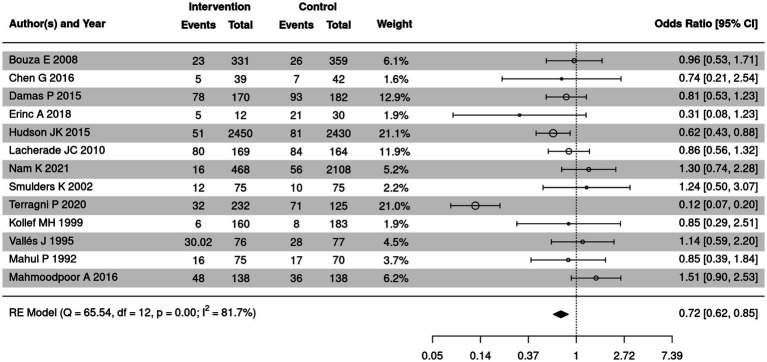
Forest plot of the comparison of fatality in SSD and control groups. CI, confidence interval; OR, odds ratio.

Fatality among patients in SSD and control groups was summarized in 13 out of 18 included articles. The meta-analysis was further performed to compare the fatality in SSD and control groups (Figure 8). Significant heterogeneity was detected among the included studies (*I*^2^ = 81.7%, *p* < 0.05); therefore, a random-effects model was used. The meta-analysis showed that the fatality in the SSD group was significantly lower than in the control group (OR = 0.72, 95% CI: 0.62–0.85, *p* < 0.0001).

### Comparison of tracheostomy in SSD and control groups

**Figure 9:**
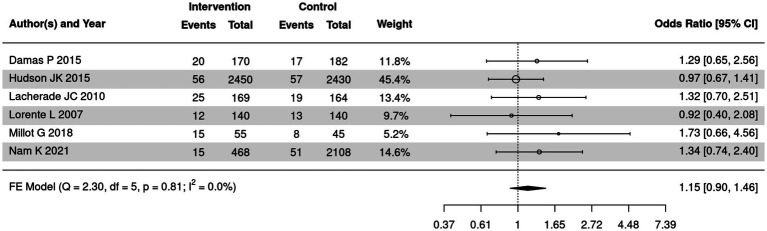
Forest plot of the comparison of tracheostomy in the two groups. CI, confidence interval; OR, odds ratio.

Tracheostomy of SSD and control groups was summarized in 6 out of 18 included articles. The meta-analysis was further employed to compare tracheostomy of the two groups (Figure 9). No heterogeneity was detected in tracheostomy of the two groups (*I*^2^ = 0%). Therefore, fixed effect model was utilized for the meta-analysis. It was revealed that no remarkable heterogeneity was detected in the two groups (OR = 1.15, 95% CI: 0.90 ~ 1.46).

### Comparison of incidence of VAP/1,000 ventilator days between SSD and control groups

**Figure 10:**
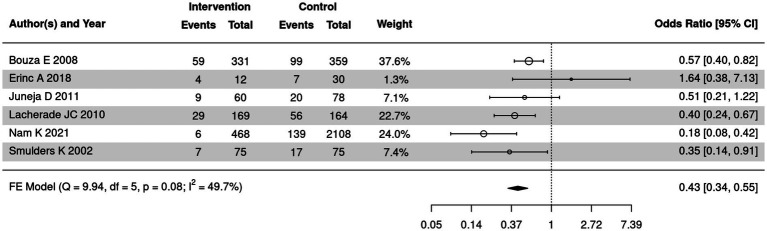
Forest plot of incidence of VAP/1,000 ventilator days of the two groups. CI, Confidence interval; OR, odds ratio.

Incidence of VAP/1,000 ventilator days of SSD and control groups was summarized in 6 out of 18 included articles. The meta-analysis was further employed to compare incidence of VAP/1,000 ventilator days of the two groups (Figure 10). Moderate heterogeneity was detected (*I*^2^ = 49.7%, *p* = 0.08); therefore, a fixed-effect model was employed. The meta-analysis indicated that the incidence of VAP/1,000 ventilator days in the SSD group was significantly lower than in the control group (OR = 0.43, 95% CI: 0.34–0.55, *p* < 0.00001).

### Comparison of the detection rate for Gram positive bacteria in SSD and control groups

**Figure 11:**
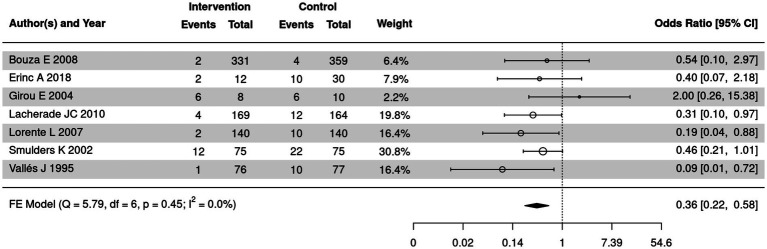
Forest plot of the comparison of the detection rate for Gram-positive bacteria in SSD and control groups. CI, confidence interval; OR, odds ratio.

The detection rate for Gram positive bacteria of SSD and control groups was summarized in 7 out of 18 included articles. The meta-analysis was further employed to compare the detection rate for Gram positive bacteria of the two groups (Figure 11). No significant heterogeneity was detected (*I*^2^ = 0%, *p* = 0.45); thus, a fixed-effect model was used. The SSD group had a significantly lower detection rate of Gram-positive bacteria than the control group (OR = 0.36, 95% CI: 0.22–0.58, *p* < 0.0001).

### Comparison of the detection rate for Gram negative bacteria in SSD and control groups

**Figure 12:**
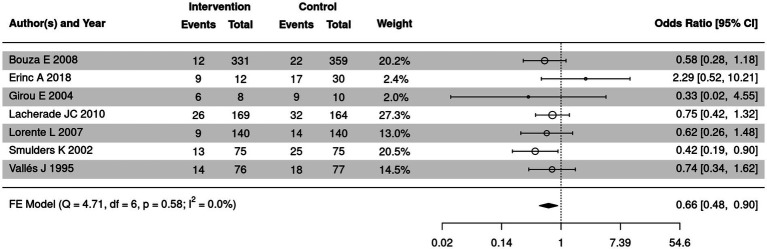
Forest plot of the comparison of the detection rate for Gram negative bacteria in SSD and control groups.

The detection rate for Gram negative bacteria of SSD and control groups was summarized in 7 out of 18 included articles. The meta-analysis was further employed to compare the detection rate for Gram negative bacteria of the two groups (Figure 12). No significant heterogeneity was detected (*I*^2^ = 0%, *p* = 0.58); a fixed-effect model was used. The results demonstrated that the detection rate of Gram-negative bacteria in the SSD group was significantly lower than in the control group (OR = 0.66, 95% CI: 0.48–0.90, *p* = 0.01).

### Analysis of publication bias

**Figure 13:**
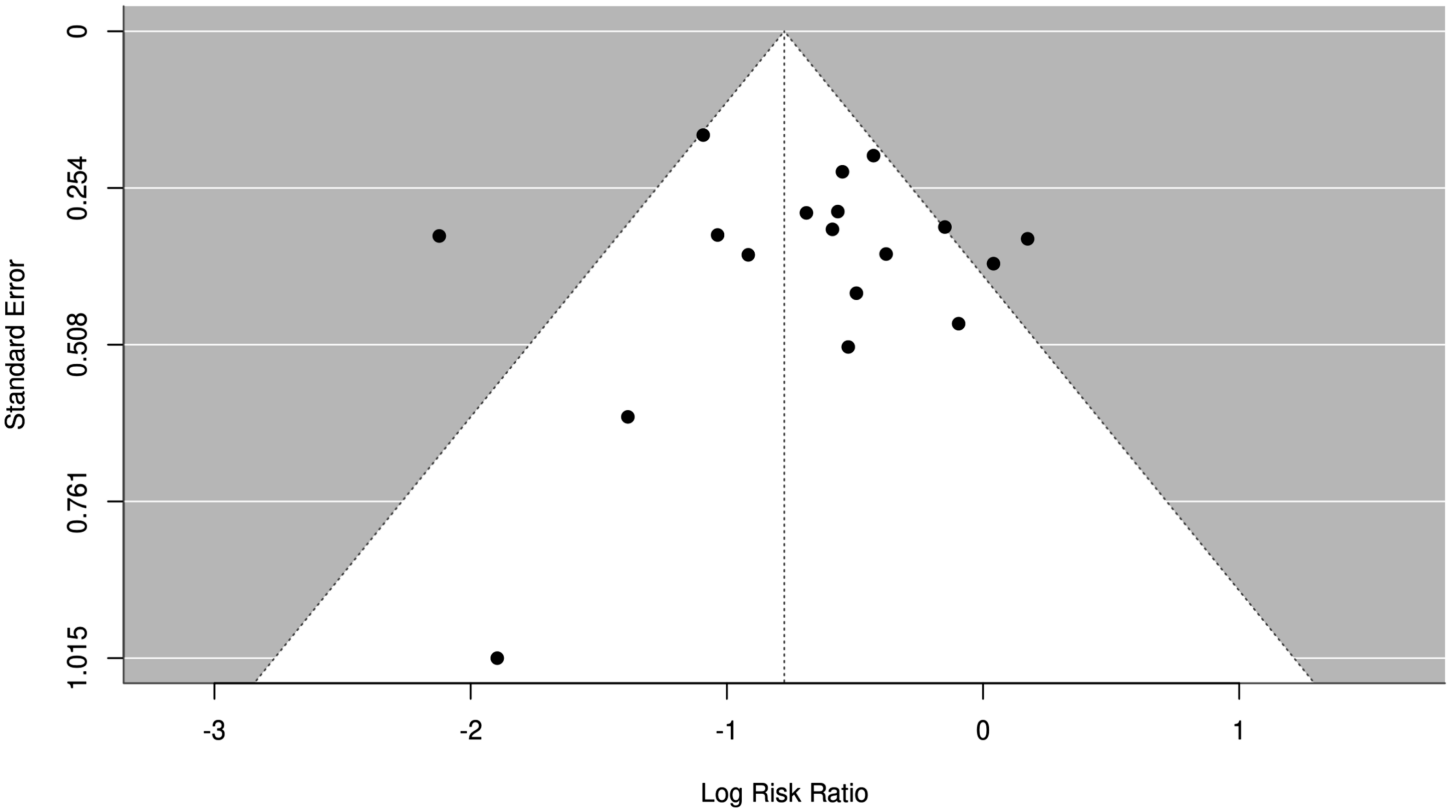
Funnel plot of VAP incidence in SSD and control groups. SE, standard error; RR, relative ratio.

**Figure 14:**
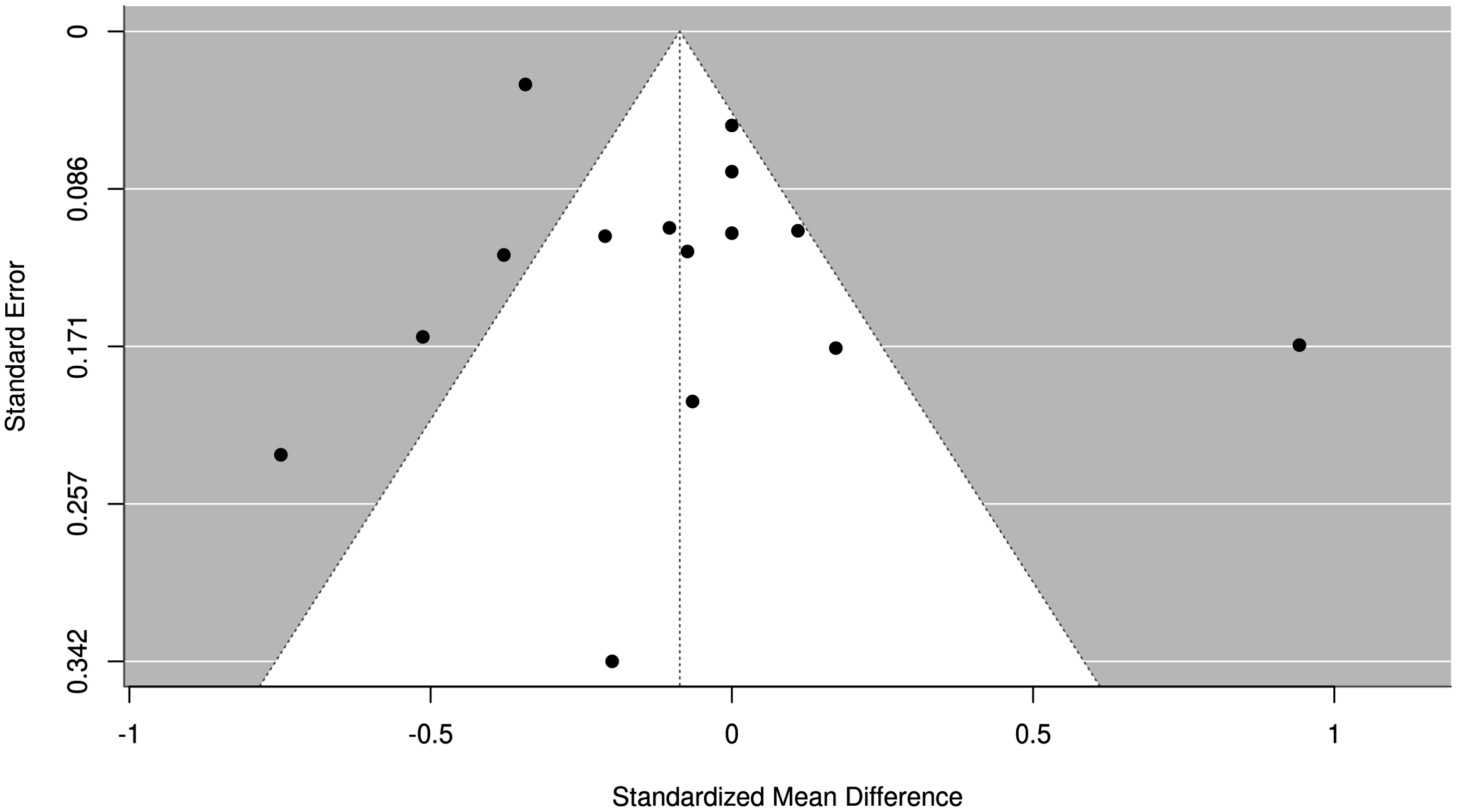
Funnel plot of the duration of ICU stay in SSD and control groups. SE, standard error; RR, relative ratio.

Inverted funnel plots were delineated to analyze the publication bias of screened articles (Figures 13, 14). It was found that the funnel plots in the included articles were symmetric and most included articles fell into the inverted funnel plots and were close to the central axis, which demonstrated that screened articles on the nursing effect of SSD on patients with ICU VAP showed low publication bias.

## Discussion

Ventilator is a commonly used device for artificial ventilation in clinical practice and it is now widely applied to surgical anesthesia, the treatment of respiratory failure, and respiratory supportive treatment. Nevertheless, the use of ventilator causes VAP. The incidence of VAP is proportional to the duration of MV and VAP prolongs ventilation time and increases fatality. According to relevant research, the fatality was only 8.5% among MV patients without VAP, while it was higher than 50% among those with VAP. Over 88% of causes of death were related to VAP infection and the occurrence of complications (17). The findings revealed that VAP incidence in SSD group was significantly lower than that in control group and remarkable heterogeneity was detected in VAP incidence in the two groups. In addition, incidence of VAP/1,000 ventilator days of SSD group was significantly lower than that of control group. The above research findings suggested that SSD nursing could markedly reduce the incidence of VAP versus routine nursing. After SSD, subglottic secretions were removed, the mucus on saccule was reduced, and the main pathway of VAP infection was cut off (18). It is well known that VAP enters lung mainly through oropharyngeal secretions and gastrointestinal reflux contents. In other words, VAP infection occurs through stomach-oropharynx-lower respiratory tract, which is divided into endogenous and exogenous VAP. High bacterial concentration in ICU and lenient visiting system as well as aseptic technique are the main factors of exogenous VAP. Aspiration of oropharyngeal secretion and gastrointestinal reflux contents and bacterial biofilm shedding are the common factors of endogenous VAP. During MV, the physiologic barriers of oropharynx and lower respiratory tract are damaged, which results in the reflux of gastrointestinal secretions and colonized bacteria. Consequently, oropharyngeal secretions and gastrointestinal reflux contents containing massive bacteria accumulate below glottis (19). The bacterial concentration in subglottic secretions and reflux contents is as high as 10^8^ ~ 10^10^ CFU/mL. A large number of bacteria flow into lower respiratory tract along tracheal wall, which leads to VAP (20). Under normal circumstances, immune system can remove pathogenic bacteria lower than 10^6^CFU/mL. Pathogenic bacteria higher than 10^7^CFU/mL destroy normal immune defense system (21). Some relevant studies show that about 90% of pathogenic bacteria of VAP are derived from pharyngeal secretions and gastrointestinal reflux contents and the bacteria cultured in VAP subglottic secretions are highly homologous to strains in gastrointestinal fluid. In addition, long duration of the retention of oropharyngeal secretions and gastrointestinal reflux contents provides an effective living environment for bacterial reproduction. Weaken blood circulation and the reduction of antimicrobial agent concentration lead to the emergence of resistant organisms and the increase of their mutants. Consequently, patients’ condition further deteriorates (22). Therefore, effective blockage of the source of VAP infection is one of the effective measures for preventing and treating VAP. SSD nursing reduces the probability of the bacteria in oropharyngeal secretions and gastrointestinal reflux contents entering lower respiratory tract, which reduces the incidence of VAP. According to a large number of studies (23, 24), SSD could notably reduce the incidence of VAP caused by Gram positive and negative bacteria and improve the prognosis for patients. What’s more, oropharyngeal secretions and gastrointestinal reflux contents were viscous so that tube blockage occurred and subglottic drainage effect was poor (21). Cotoia et al. (25) showed that intermittent rinsing fluid combined with CASS could the viscosity of secretions and reflux contents to improve subglottic drainage effect.

According to the research findings, no remarkable heterogeneity was detected in MV duration, length of hospital stay, and length of ICU stay of SSD and control groups. It suggested that SSD could not shorten MV duration and length of hospital stay and reduce fatality, which was different from the study outcome obtained by Chair et al. (26) and Ghoochani Khorasani et al. (27). The difference might be caused by various clinical variables. There might be other potential factors related to MV duration, length of hospital stay, and fatality in different studies. The lack of significant difference in MV duration and overall hospital stay suggests that while SSD effectively prevents VAP, the ultimate length of stay for critically ill patients is likely multifactorial, driven by underlying admission diagnoses, multi-organ dysfunction, and other ICU-acquired complications that SSD cannot address. The findings revealed that the detection rates for Gram positive and negative bacteria in SSD group were both significantly lower than those in control group, which indicated that SSD could reduce the detection rates for the above bacteria. The decline in the detection rates was of great significance for preventing VAP and reducing its incidence. The existing research results demonstrate that Gram negative bacteria account for 83.9% in the secretions on saccus and lower respiratory tract among MV patients and the infection rate of *Pseudomonas aeruginosa* is the highest (26.9%). In contrast, the proportion of Gram positive bacteria is only 12.0%, which mainly consist of *Staphylococcus epidermidis* and *Staphylococcus aureus* (28). Klompas (29) showed that Gram negative bacterium was the main colonized bacterium in the airways of MV patients. It was inhaled by pharyngeal secretions into gastrointestinal tract, which resulted in bacterial retrogradation followed by VAP. Maertens et al. (30) found that SSD could notably reduce the incidence of VAP caused by Gram positive coccus and *Haemophilus influenzae*.

## Conclusion

This meta-analysis demonstrates that implementing subglottic secretion drainage significantly reduces the incidence of ventilator-associated pneumonia and decreases the detection rates of both Gram-positive and Gram-negative bacteria in ICU patients. While it may not independently reduce overall MV duration or hospital length of stay, SSD is a highly effective, targeted intervention for airway management and infection control. Routine implementation of SSD should be strongly considered as part of standard VAP prevention bundles.

### Limitations

Nonetheless, there were still some limitations in this research. The grouping methods were not clarified in some included articles in the meta-analysis. In addition, the number of included cases was small in a few articles. There were differences in specific interventions, follow-up, and the selection of outcome indicators, which resulted in the small number of included articles with some outcome indicators and possible bias in the results. In follow-up research, the application values of SSD in the nursing for patients with ICU VAP will be further verified based on clinical trials. In conclusion, the research results provided some referable basis for the prevention and nursing of ICU VAP.

## Data Availability

The original contributions presented in the study are included in the article/supplementary material, further inquiries can be directed to the corresponding author.
